# Supplementary material for: Crystal structure of an archaeal CorB magnesium transporter
Source: Nat Commun. 2021 Jun 29;12:4028. doi: 10.1038/s41467-021-24282-7 (PMC8242095; doi:10.1038/s41467-021-24282-7)
Supplement: Supplementary file 1 — Supplementary Information [file 41467_2021_24282_MOESM1_ESM.pdf]

Supplementary Information

Crystal structure of an archaeal CorB magnesium transporter

Yu Seby Chen[1], Guennadi Kozlov[1], Brandon E. Moeller[2], Ahmed Rohaim[3,4], Rayan Fakih[1], Benoît Roux[3], John E. Burke[2,5], Kalle Gehring[1*]

[1]Department of Biochemistry & Centre de Recherche en Biologie Structurale, McGill University, Montréal, QC, Canada

[2]Department of Biochemistry and Microbiology, University of Victoria, Victoria, BC, Canada

[3]Department of Biochemistry and Molecular Biology, University of Chicago, Gordon Center for Integrative Science, Chicago, Illinois, USA

[4]Department of Biophysics, Faculty of Science, Cairo University, Egypt.

[5]Department of Biochemistry and Molecular Biology, The University of British Columbia, Vancouver, British Columbia, Canada

[*]Correspondence: kalle.gehring@mcgill.ca

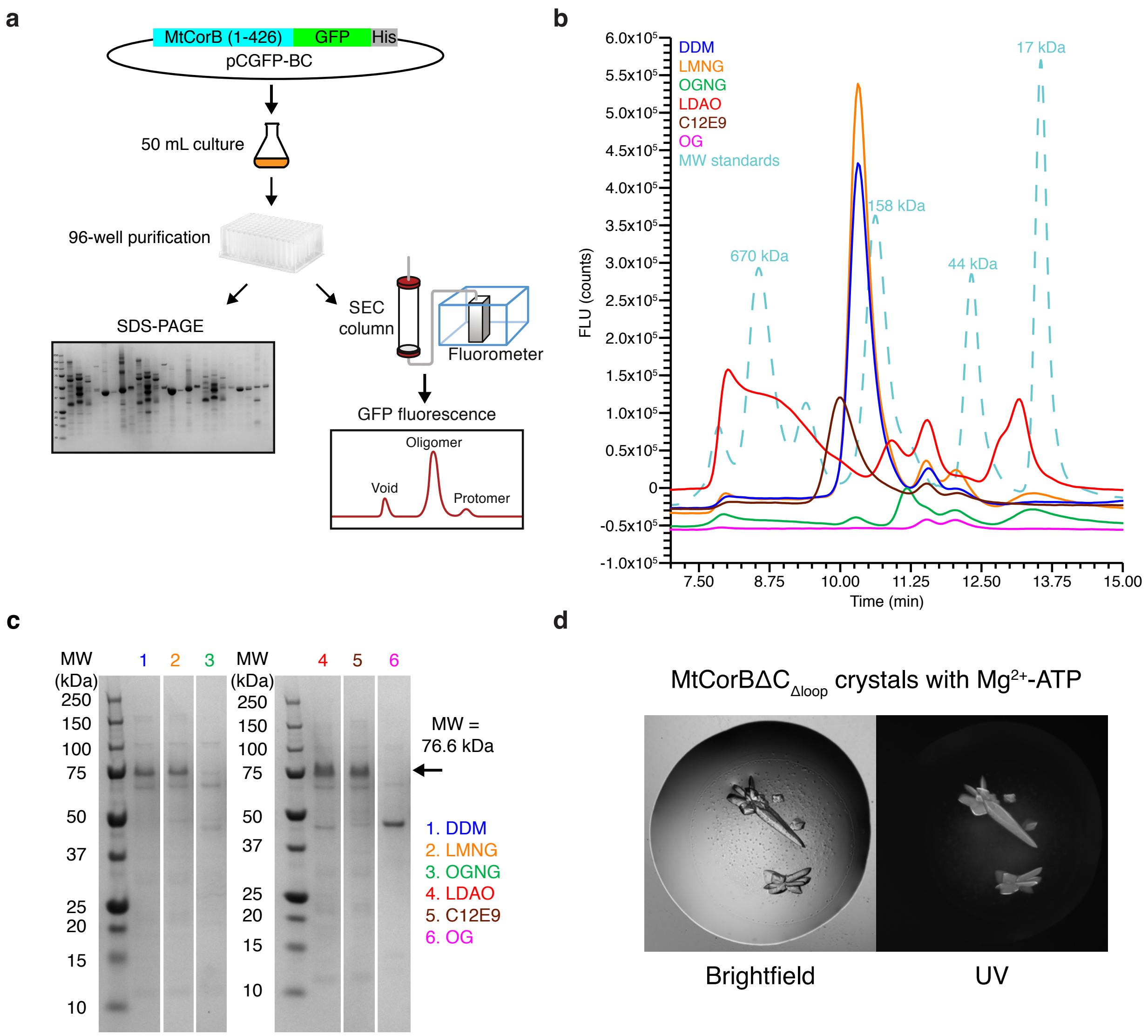

Supplementary Figure 1. Detergent screening and crystallization of MtCorB. a, Schematic of high-throughput screening process. **b**, Size-exclusion chromatography (SEC) profile of GFP-MtCorB purified in different detergents. The dashed line shows molecular weight (MW) standards. **c**, SDS-PAGE analysis of GFP-MtCorB purified in 6 detergents. This experiment was only performed once. **d**, MtCorBΔC$_{\Delta loop}$ crystals in complex with Mg^{2+}-ATP taken with brightfield (*left*) and UV (*right*) camera.

```
            TM1                                α1            α2
Archaea     MVVIDLLIVEVVLFIAALLFSGFFSSSEVALISITRAKVHALQSQGRKG----AKALDTLKRST-DA  62
Bacteria    DSMLADLLWQWIGLAVLLLTSGFASMSETVMMAANRYRLKARADNGERG----AQLAAALTAHP-ER  67
Plant       SQVLKVLREQGLILAVLLGLSAFFSMAETSITTLWPWKVRELAEKEPE-----NGVFRMLRSDV-TR  207
Yeast       VGDEASVTTYYIISIILVLLGGVFAGLTLGLMGQDEVYLKVISTSGSNSEKKLAKRVLDLISRGKHW  121
Roundworm   KEYFLPLPLQIACIGFLLCLSALFSGLTLGLMSLTPQELELVIKSGAIKEQKCAAKILPVRKKG-NL  260
Fruit fly   FEPLIPVWLAIIIIVTCLGFSALFSGLNLGLMSMDRTELKILRNTGTEKEKKYASKIAPVRDQG-NY  245
Zebrafish   KKFLLPFWLQVIFIAMLLCLSGMFSGLNLGLMALDPMELRIVQNCGTEKEKHYAKAIEPVRSQG-NY  257
Human       KKFLLPFWLQVIFISLLLCLSGMFSGLNLGLMALDPMELRIVQNCGTEKEKNYAKRIEPVRRQG-NY  314
               .             :  ... :     :       :.                   :

            TM2                          TM3
Archaea     IQITTLIGSTIANVAVASLATAIGITLYGN--LGIAVGLVVAAVLVLVFGEIGPKMYASRYTEELAL  127
Bacteria    MLSVILLVNNAVNVGAATLASVITIELFGQNETMLAVGSFVLTFLILVFSEITPKVIGARYADLLAP  134
Plant       FLTTILIGTTVVNIAATALVTKAATAIFGE--AGVSAATGVMTVAILLLTEITPKSVAVHNAQEVAR  272
Yeast       VLVTLLLSNVITNETLPIVLDRCL--------GGGWQAVVSSTILIVIFGEIIPQSVCVKYGLQVGA  180
Roundworm   LLCSLLLGNVIVNSAISILMGEL---------TTGIYALIGSTMGIVIFGEILPQSICVKKGLEVGA  318
Fruit fly   LLCSILLGNVLVNSTFTILLDGL---------TSGLFAVIFSTLAIVLFGEITPQAVCSRHGLAIGA  303
Zebrafish   LLCSLLLGNVLVNTTLTILLDDIA--------GSGLIAVVVSTIGIVIFGEIVPQAICSRHGLAVGA  316
Human       LLCSLLLGNVLVNTTLTILLDDIA--------GSGLVAVVVSTIGIVIFGEIVPQAICSRHGLAVGA  373
            .    *: .  .*     :                  .    :. :::: ** *:    :    :.

            JM                                         α3
Archaea     RVSRPILFFSKLLYPVLWVTDRIEQQF---AFRPGVTEP-VVTEEEIKEWIDVGEEEGTI--EEEER  188
Bacteria    YIAYPLTAILRLVGPVVDFVNLFVKGLLWLLRLPRRATPQAPSLEELRSLVLESRVLR----SEKHR  197
Plant       IVVRPVAWLSLILYPVGRVVTYLSMGILKILGLKGRSEP-YVTEDELKLMLRGAELSGAI--EEEEQ  336
Yeast       FFCPFVLVLMYLMYPVAYPIATLLDYML------GEDHGTMYKKSGLKTLVTLHRTMGVERLTKDEV  241
Roundworm   HTISITQLFIFLTFPIAWPVSKLLDCLL------GDEYQ-AYDRKRLMELIKMSITDNGQ--VSNEL  376
Fruit fly   KTILVTKTVMAITAPLSYPVSRILDKLL------GEEIGNVYNRERLKELVRVTNDVNDL--DKNEV  362
Zebrafish   NTIFLTKFFMILTFPASYPVSKLLDHVL------GQEIGTVYNREKLLEMLRVTDPYNDL--VKEEL  375
Human       NTIFLTKFFMMMTFPASYPVSKLLDCVL------GQEIGTVYNREKLLEMLRVTDPYNDL--VKEEL  432
                    .  :  *       :   .               .   :   :            ...

            α4        αA1  αA2        α5            β1            β2
Archaea     DMLYSVLRFGDTTAREVMTPRVDVVMIEDTATLESA-LAIFNETGFSRIPVYH-ERIDNIVGLLNVK  253
Bacteria    DVLLKLFDLERITVADVMIPRQAIEFLDLTDDEETL-RAQLATAYHTRLPVIE-GNPDEVLGILHVR  262
Plant       DMLENVLEIKDTHVREVMTPLVDVVAIDGSGSLVDF-HNFWVTHQYSRVPVFE-QRIDNIVGIAYAM  401
Yeast       TIISAVLDLKAKRVEEIMTPIENVFTMSADTILDDKTVEKIFNSGFSRIPIFLPNEPNNFIGMLLVR  308
Roundworm   KIAVGAMEIADKVVKDVMTKIEDVFMLPDTTVLNAKTVMEIVKMGYTRIPVYQYGDKNNVTDMLFVK  443
Fruit fly   NIISGALELRKKTVADVMTHINDAFMLSLDALLDFETVSEIMNSGYSRIPVYD-GDRKNIVTLLYIK  428
Zebrafish   NIIQGALELRTKTVEDVMTPLRDCFMISGDAILDFATMSEIMESGYTRIPVYE-GERCHIVDLLFVK  441
Human       NIIQGALELRTKTVEDVMTPLRDCFMITGEAILDFNTMSEIMESGYTRIPVFE-GERSNIVDLLFVK  498
             :    : :    . ::*        :                  .:*:*:       ..  :

            α6             αB      β3      α7           β4
Archaea     DVFSAVFRQQ--TSATIRDL-MYEPYFIPESKKIDELLKELQVKK-QHMAVVLDE---------YGS  307
Bacteria    QLLAETLTSGFSR--EAIRRSLSPPYFVPEETNAMTQLQFFQEHH-QRLALVVDE---------YGE  317
Plant       DLLDYVPKGKLLESTTVVDMAHKPAFFVPDSMSVWNLLREFRIRK-VHMAVVLNE---------YGG  458
Yeast       VLISYDPDDCLPI-SHFP---LATLPETSPNTSCLNILNYFQEGK-AHMCVVSKE------PGSSHG  364
Roundworm   DLALLDPDDNFTV-KTVCGYHKHPVKFVMNDTPLPNLLEAFKKGE-GHLAMVKRLINTDDKHDPSYV  508
Fruit fly   DLAFVDTDDNTPL-KTLCEFYQNPVHFVFEDYTLDIMFNQFKEGTIGHIAFVHRV-NNEGDGDPFYE  493
Zebrafish   DLAFVDPDDCTPL-KTITKFYSHPLHFVFNDTKLDAMLEEFKKGK-SHLAIVQRV-NNEGEGDPFYE  505
Human       DLAFVDPDDCTPL-KTITKFYNHPLHFVFNDTKLDAMLEEFKKGK-SHLAIVQRV-NNEGEGDPFYE  562
             :                            .      :. ::     ::..*

            β5   α8
Archaea     FAGIVTVEDMLEELV  322
Bacteria    LQGLVTLDDIIEEMV  332
Plant       TIGIVTLEDVVEEIV  473
Yeast       AIGVLTLEDVIEELI  379
Roundworm   LVGVVTLEDIVEEIL  523
Fruit fly   TVGLVTLEDVIEELI  508
Zebrafish   VLGIVTLEDVIEEII  520
Human       VLGIVTLEDVIEEII  577
              *::*::*::**::
```

- residues involved in coordinating Mg^{2+} ion
- polar residues in the cavity of inward-facing conformation
- polar residues in outward-facing conformation
- kink-inducing residue in JM helix
- ATP-binding site residues

Supplementary Figure 2. Sequence alignment of CorB/CNNM orthologs from eight representative species. The listed CorB/CNNM orthologs and their UniProt accession numbers are: MtCorB (*Methanoculleus thermophilus*; A0A1G8XA46), TtCorB (*Tepidiphilus thermophilus*; A0A0K6IWT9), CBSDUFCH2 (*Arabidopsis thaliana*; Q84R21), MAM3 (*Saccharomyces cerevisiae*; Q12296), cnnm-1 (*Caenorhabditis elegans*; A3QM97), UEX (*Drosophila melanogaster*; A0A0B7P9G0), cnnm2a (*Danio rerio*; A2ATX7), and CNNM2 (*Homo sapiens*; Q9H8M5). Secondary structure corresponds to the crystal structure of MtCorBΔC. Highlighted residues: residues involved in coordinating Mg^{2+} ion (*magenta*), polar residues in the cavity of inward-facing conformation (*blue*), polar residues in outward-facing conformation (*green*), kink-inducing residue in JM helix (*yellow*), and ATP-binding site residues (*orange*).

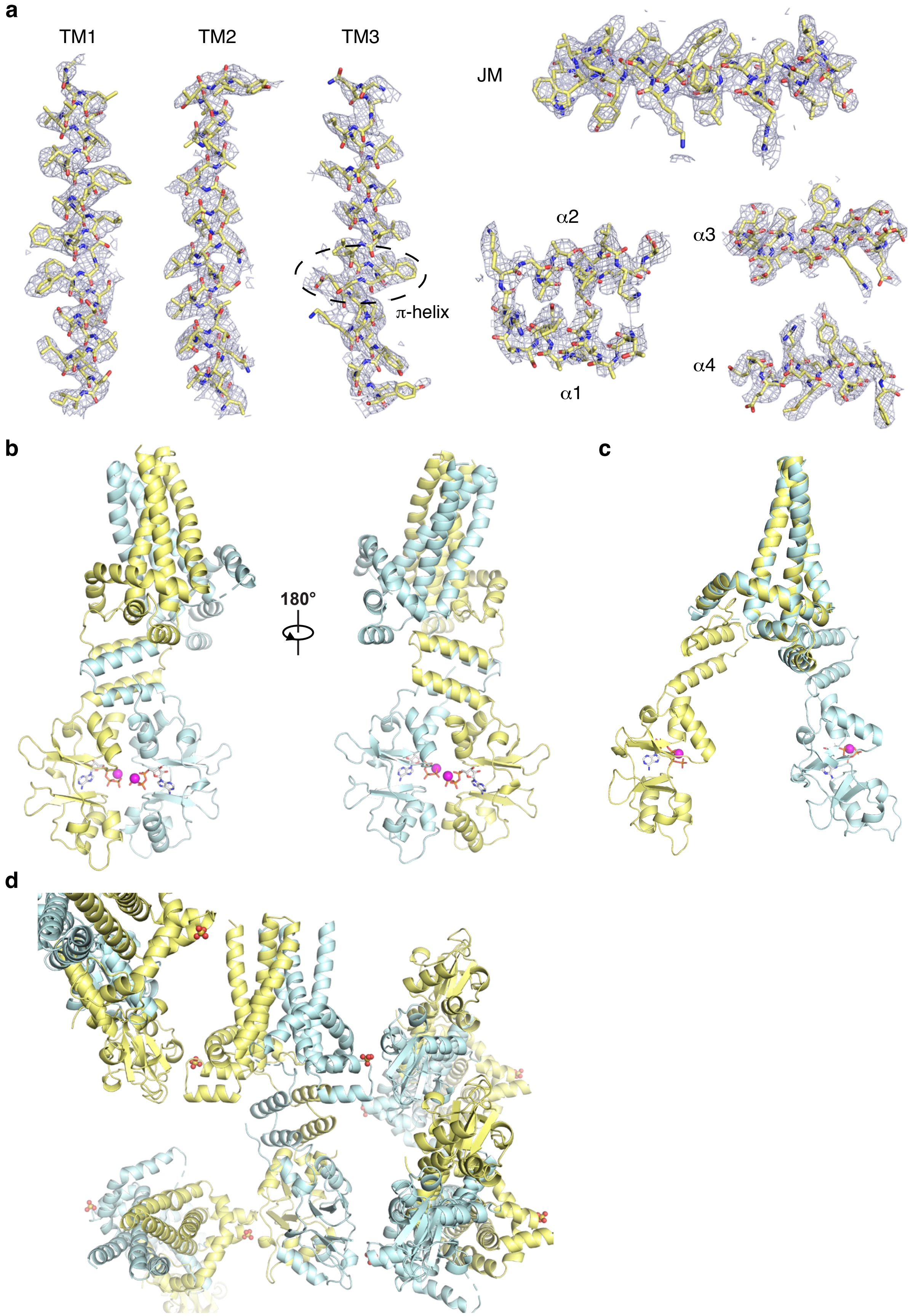
a
TM1
TM2
TM3
JM
π-helix
α2
α1
α3
α4
b
180°
c
d

Supplementary Figure 3. Overall structure of MtCorBΔC bound to Mg^{2+}ATP.

a, Representative simulated annealing $2F_o$-F_c composite omit map for TMD and AHB, contoured at 1.0 σ. **b**, Front and rear view of the MtCorBΔC homodimer showing asymmetry between TMD and cytosolic domains. **c**, Overlay of the TMD of the two protomers. **d**, Crystal packing is assisted with a sulfate molecule.

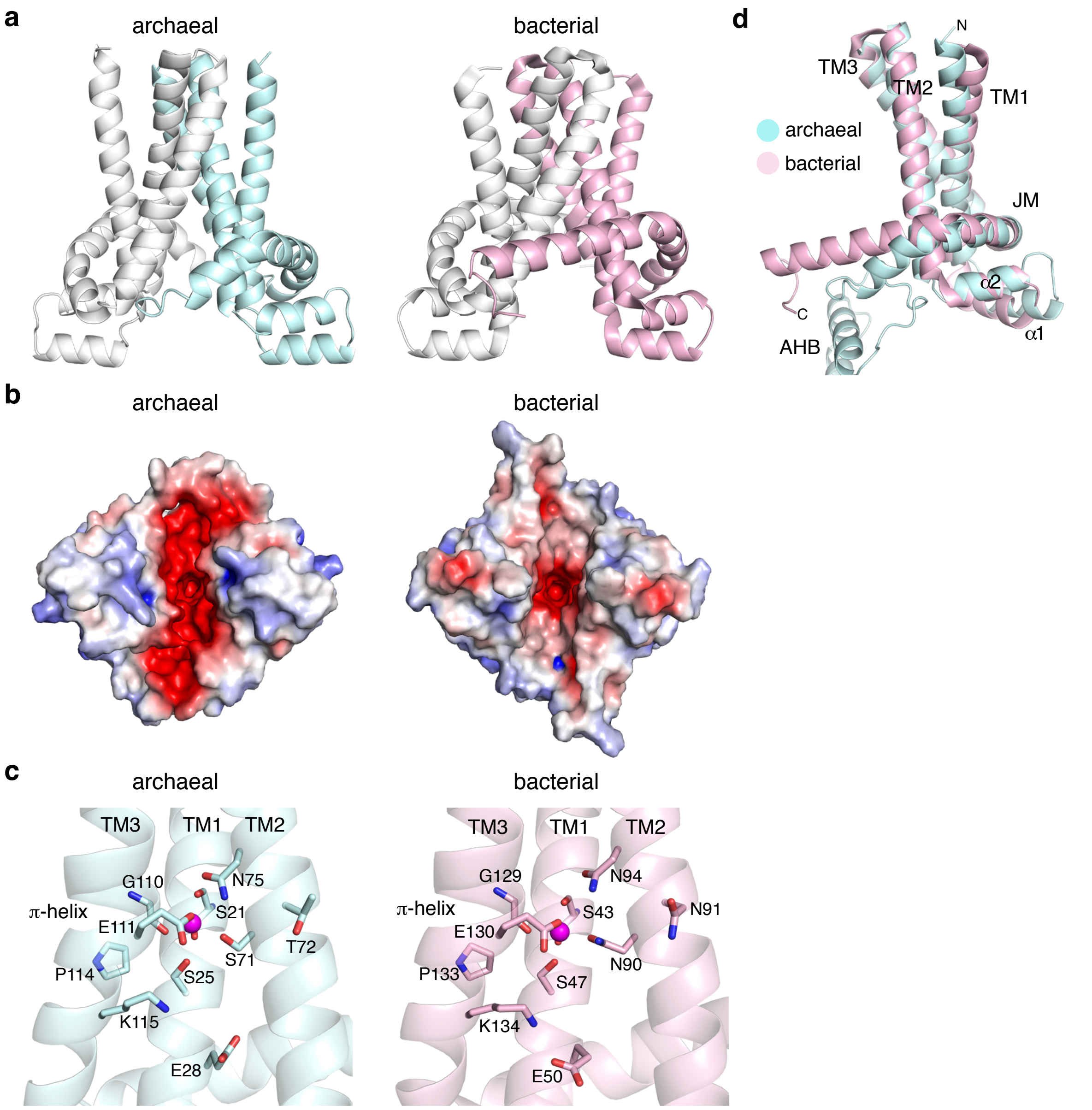

Supplementary Figure 4. Structural comparisons of the TMD of archaeal (this work) and bacterial (PDB: 7CFF) CorB proteins. a, TMD homodimerizes in both archaeal and bacterial CorB proteins. **b**, Electrostatic surface potential analysis (±5 kT e^{-1}) shows conservation of negatively charged cavity in both archaeal and bacterial CorB proteins from an intracellular view. **c**, Conservation of the polar residues in the Mg^{2+} binding site and π-helical turn of archaeal and bacterial CorB proteins. **d**, Structural overlay of the TMD of archaeal and bacterial CorB proteins showing distinct differences in the length and orientation of the TM1 and JM helices.

a

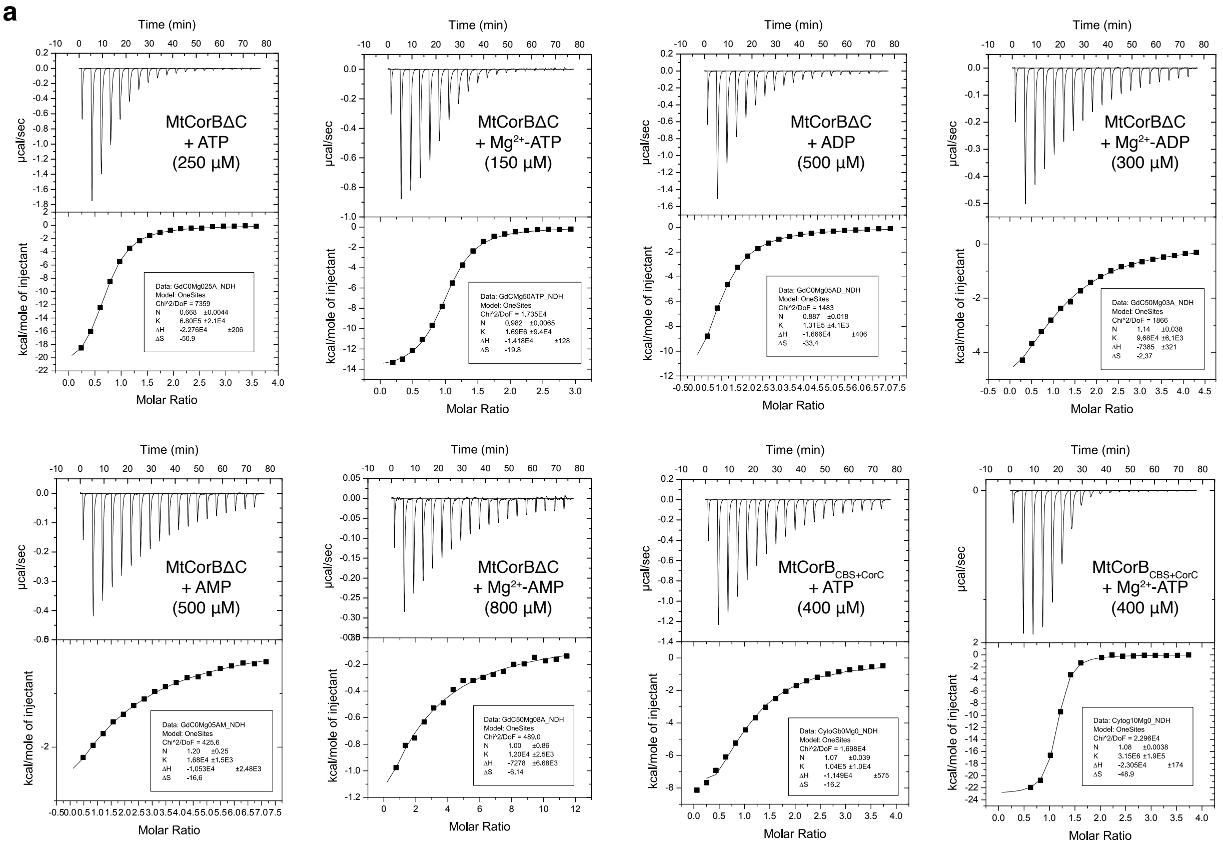

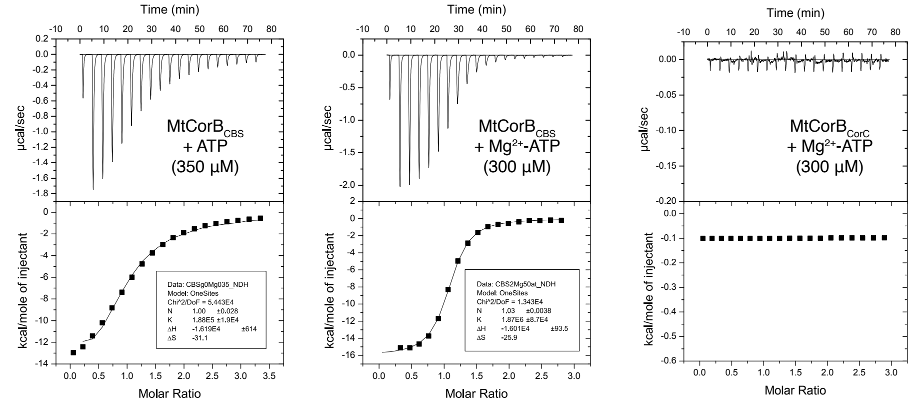

b

Construct	K_d (μM)	
	no Mg^{2+}	with Mg^{2+}
TMD+CBS	1.47 ± 0.15	0.59 ± 0.06
CBS+CorC	9.6 ± 1	0.32 ± 0.03[1]
CBS	5.3 ± 0.5	0.53 ± 0.05
CorC	-[2]	no binding

[1]Done with 10 mM instead of 50 mM $MgCl_2$
[2]Not tested

Supplementary Figure 5. Raw ITC thermograms. a, ITC thermograms showing various MtCorB constructs binding to adenosine nucleotides (concentrations indicated in parentheses) in absence or presence of 50 mM $MgCl_2$. **b**, Table summarizing the affinities of different MtCorB constructs binding to ATP in presence or absence of 50 mM $MgCl_2$.

a

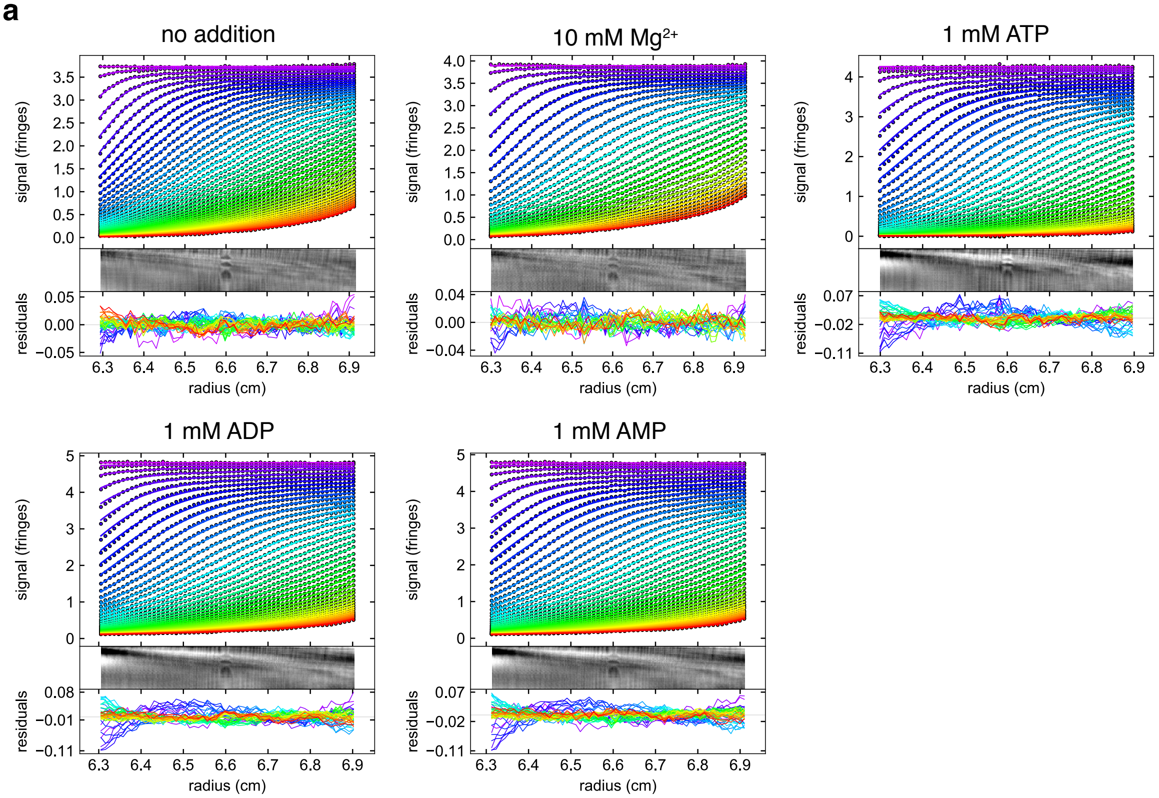

b

	Sedimentation coefficient (S)		Estimated MW (kDa)		RMSD	Theoretical MW (kDa)	
Condition	Peak 1	Peak 2	Peak 1	Peak 2		Monomer	Dimer
no addition	1.84	-	16.2		0.0049		
10 mM Mg^{2+}	1.78	-	16.0	-	0.0042		
1 mM ATP	-	2.49	-	32.0	0.0045	14.5	29.0
1 mM ADP	-	2.40	-	30.4	0.0044		
1 mM AMP	-	2.44	-	30.9	0.0050		

Supplementary Figure 6. Summary of SV-AUC results. a, Sedimentation velocity AUC profiles of $MtCorB_{CBS}$ in presence of various ligands. Interference of the sample are plotted against the radial position in the cell. One in every 75 scans is plotted. **b**, Summary of experimental sedimentation coefficients and estimated molecular weights.

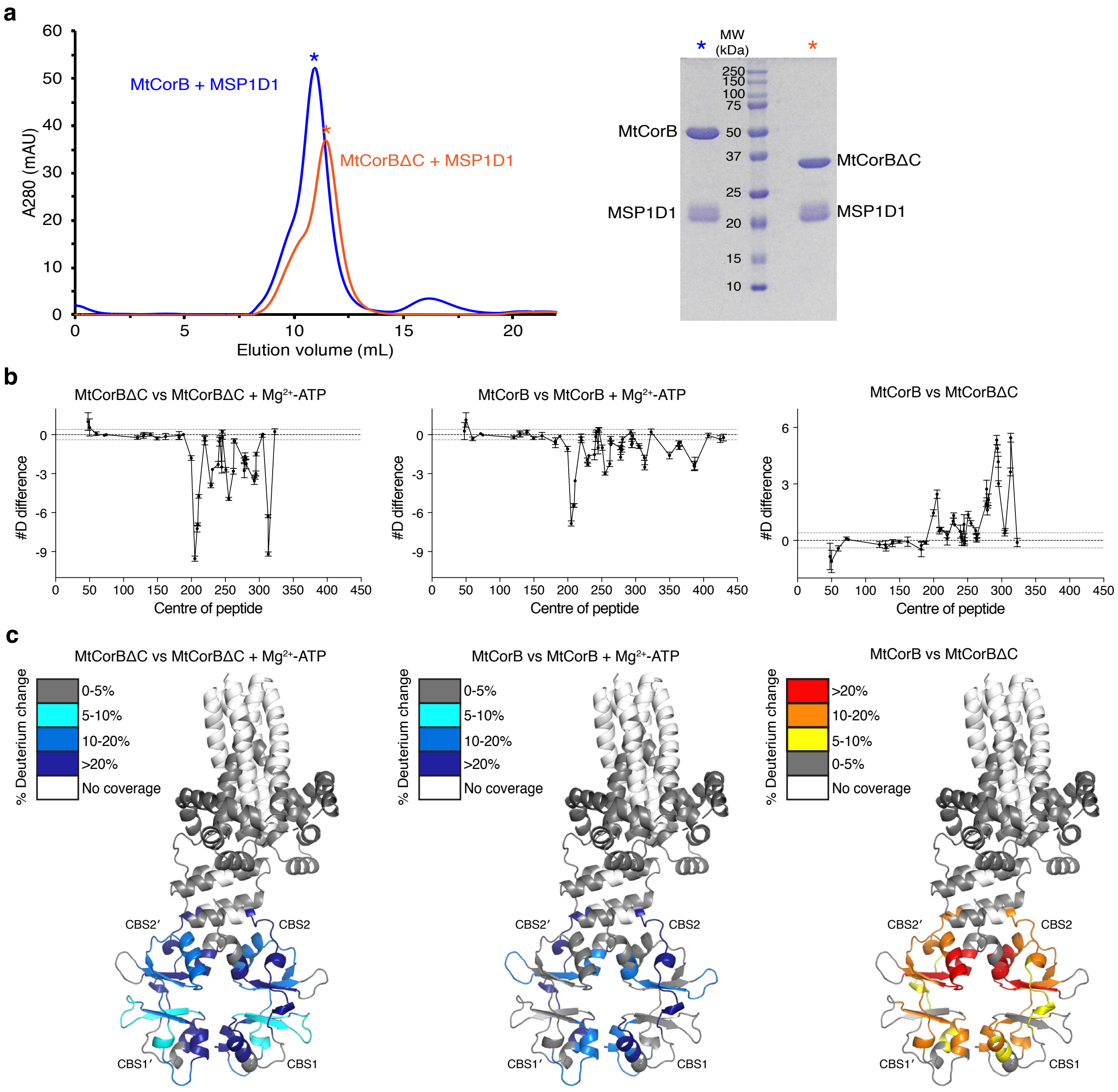

Supplementary Figure 7. Reconstitution of MtCorB into MSP1D1 nanodiscs and HDX-MS analyses. a, SEC profile and SDS-PAGE analysis of MtCorB and MtCorBΔC reconstituted in MSP1D1 nanodiscs. The experiment was repeated twice with similar results. **b**, HDX-MS analysis of three sets of experiments. The sum of the # of deuterons protected from across all timepoints is shown. Each point represents a single peptide, with them being graphed on the x-axis according to its central residue. The data points represent mean ± SD (n = 3 independent measurements). **c**, Mapping of deuteration onto the structure of MtCorBΔC. Regions that showed

significant decreases or increases in exchange (defined as >5%, 0.4 Da, and a two-tailed Student's *t*-test $p<0.01$) are colored in blue or red respectively. The source data are provided as a Source Data file.

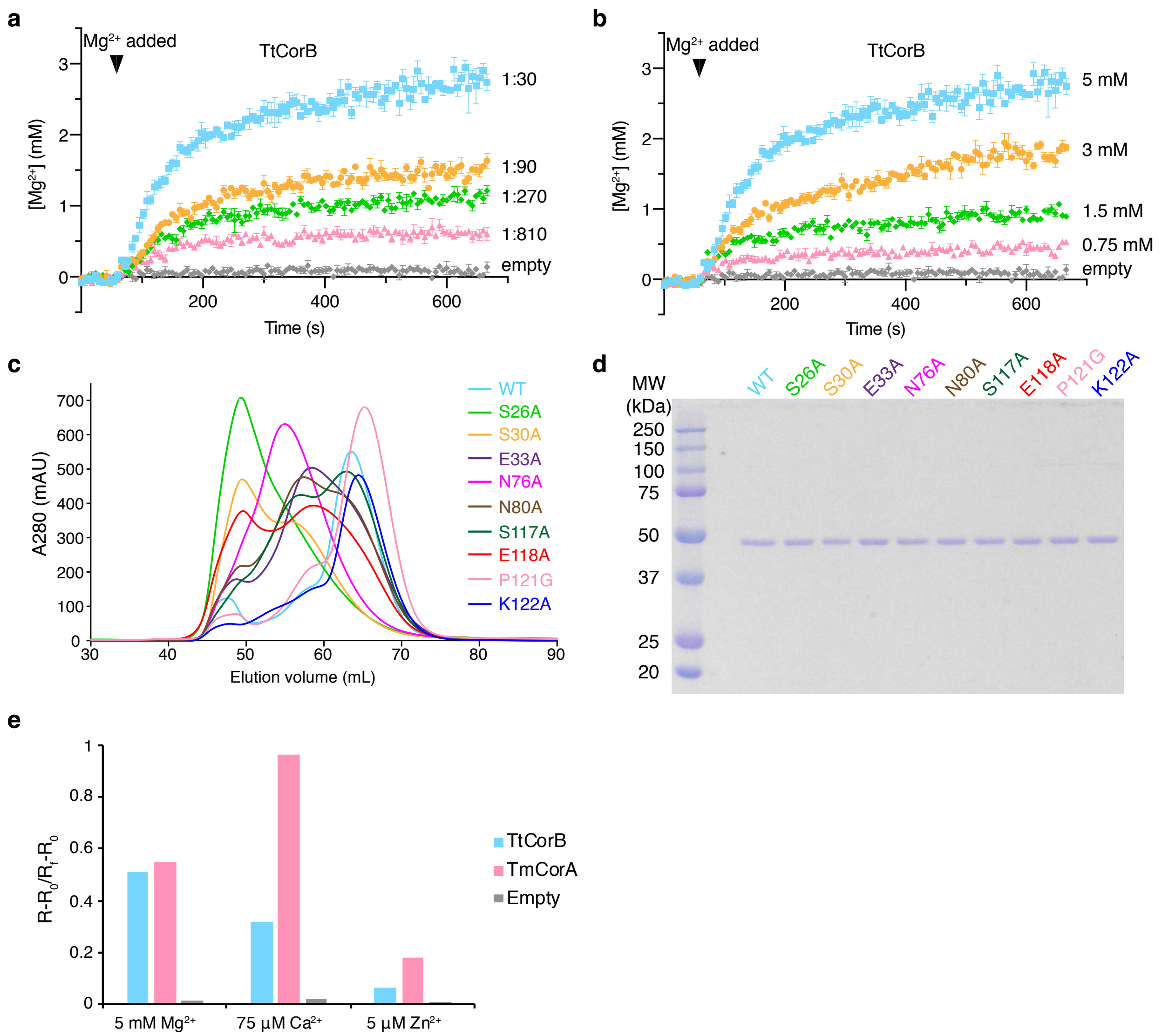

Supplementary Figure 8. Liposome mag-fura-2 Mg^{2+} transport assay of TtCorB proteins. **a,** TtCorB-incorporated proteoliposome (protein/lipid ratios of 1:30 to 1:810) shows protein-dependent Mg^{2+} transport. The data points represent mean ± SEM (n = 3 independent measurements). **b**, The TtCorB-mediated Mg^{2+} transport depends on external $MgCl_2$ concentration. The data points represent mean ± SEM (n = 3 independent measurements). **c**, SEC profiles of TtCorB mutants. The experiment was repeated twice with similar results. **d**, SDS-PAGE analysis of proteoliposomes reconstituted with TtCorB mutants (protein/lipid ratios of 1:30). **e**, Comparison of ion transport by TtCorB and TmCorA proteoliposomes 10 min after

addition of the indicated ions. The relative change in fluorescence is plotted with R = ratio of fluorescence with excitation at 330 nm /369 nm, R_0 = fluorescence ratio before addition of cations (final concentrations are shown), and R_f = fluorescence ratio after lysing liposomes with Triton X-100.

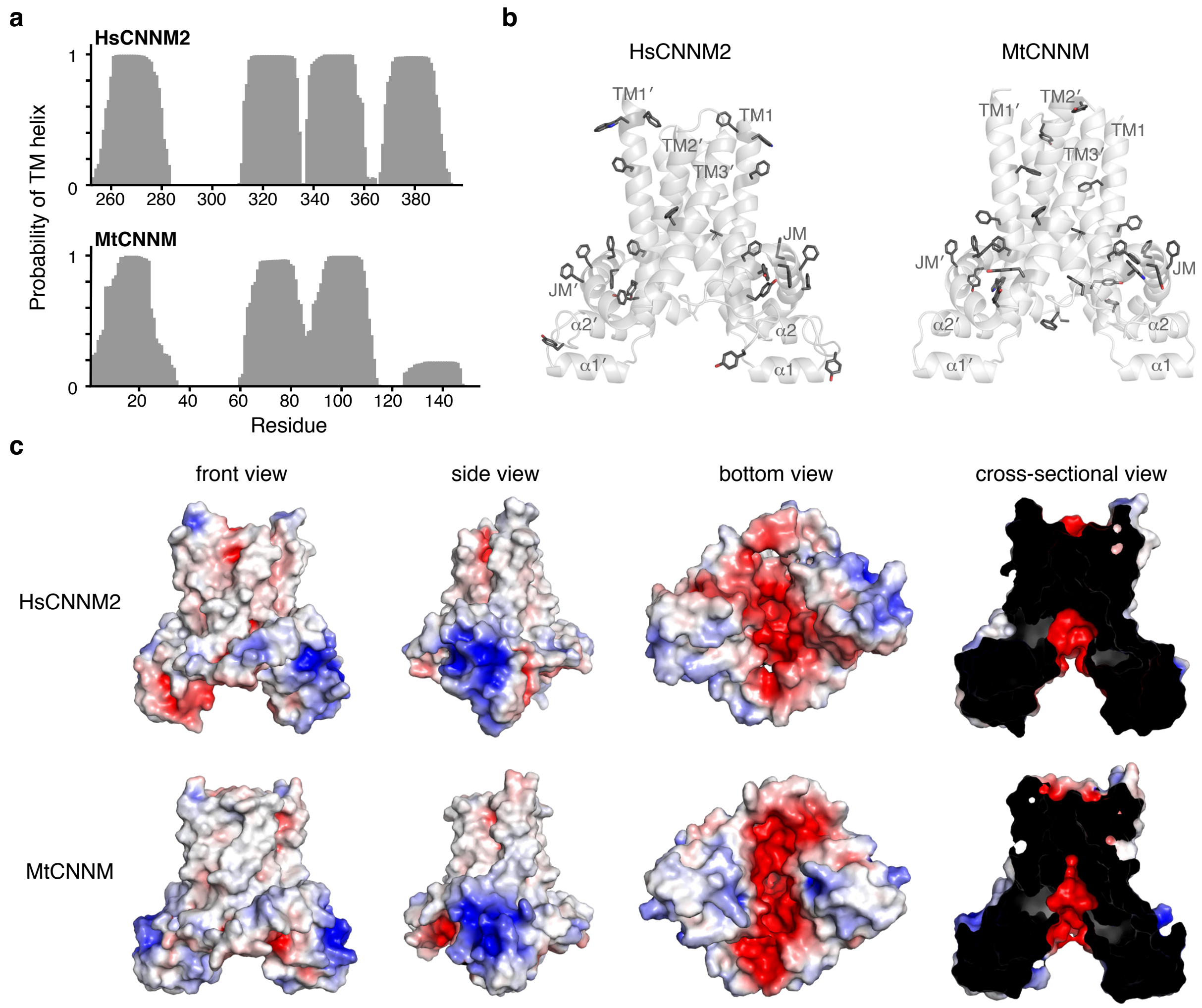

Supplementary Figure 9. Analysis of HsCNNM2 model structure. a, Prediction of transmembrane helices in human CNNM2 and archaeal MtCorB with TMHMM server. HsCNNM2 is predicted to have four TM helices. **b**, Clustering of aromatic residues at the phospholipid-solvent interface and JM helix. **c,** Comparison of the electrostatic surfaces of human and archaeal TMD (±5 kT e^{-1}).

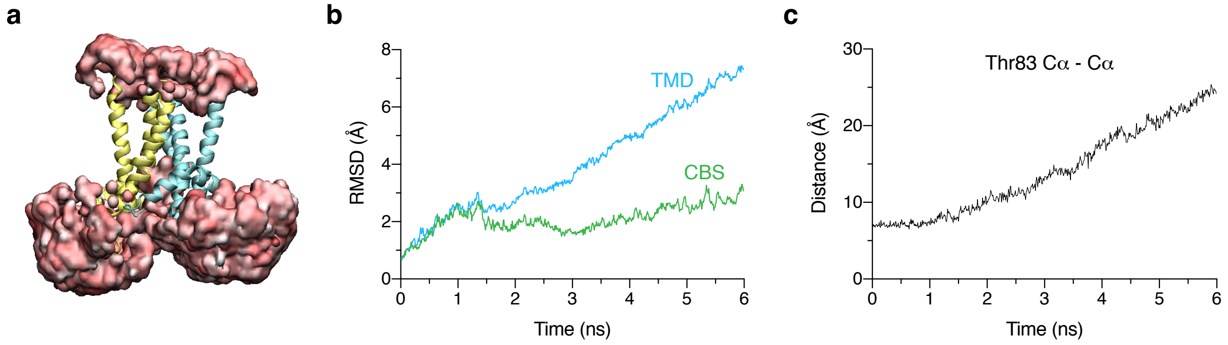

Supplementary Figure 10. Molecular dynamics simulations of MtCorB. a, Hydration analysis of TMD showing an inward-facing, solvent-exposed cavity. **b**, Structural deviations of TMD and CBS-pair domain during the targeted MD simulation of inward-facing to outward-facing conformation. **c,** Opening of the dimerization interface as shown by increased Cα distances between Thr83 of both protomers.

Supplementary Table 1. Mutagenic primers used in the study.

Construct	Forward Primer	Reverse Primer
MtCorB Δ323-426	catgctggaagaactggttctcgagcaccaccaccacc accactg	cagtggtggtggtggtggtgctcgagaaccagttcttcca gcatg
MtCorB Δ259-262	gatgtgttctctgcgcagacctccgctacc	ggtagcggaggtctgcgcagagaacacatc
MtCorB R235L	gaaaccggcttctctcttatcccggtttaccac	gtggtaaaccgggataagagagaagccggtttc
TtCorB S26A	gttctgctgctgaccgccggtttcgcgtccatg	catggacgcgaaaccggcggtcagcagcagaac
TtCorB S30A	gaccagcggtttcgcggccatgtctgaaaccg	cggtttcagacatggccgcgaaaccgctggtc
TtCorB E33A	gtttcgcgtccatgtctgcaaccgttatgatggcagc	gctgccatcataacggttgcagacatggacgcgaaac
TtCorB N76A	gagcgttatcctgctggttgccaacgcggttaacgttgg	ccaacgttaaccgcgttggcaaccagcaggataacgctc
TtCorB N80A	ctggttaacaacgcggttgccgttggtgctgcgacc	ggtcgcagcaccaacggcaaccgcgttgttaaccag
TtCorB S117A	cttcctgatcctggtgttcgccgaaatcaccccgaaag	ctttcggggtgatttcggcgaacaccaggatcaggaag
TtCorB E118A	gatcctggtgttcagcgcaatcaccccgaaagttatc	gataactttcggggtgattgcgctgaacaccaggatc
TtCorB P121G	gtgttcagcgaaatcaccgggaaagttatcggagcgcg	cgcgctccgataactttcccggtgatttcgctgaacac
TtCorB K122A	cagcgaaatcaccccggcagttatcggagcgcgttac	gtaacgcgctccgataactgccggggtgatttcgctg
