# Supplementary material for: Crystal structure of an archaeal CorB magnesium transporter
Source: Nat Commun. 2021 Jun 29;12:4028. doi: 10.1038/s41467-021-24282-7 (PMC8242095; doi:10.1038/s41467-021-24282-7)
Supplement: Supplementary file 7 — Description of Additional Supplementary Files [file 41467_2021_24282_MOESM7_ESM.docx]

Description of additional supplementary information

Title: Supplementary Movie 1

Description: MtCorBΔC undergoes drastic conformational changes upon Mg^2+^-ATP binding.

Title: Supplementary Movie 2

Description: Unbiased MD simulations of Mg^2+^-ATP-bound MtCorBΔC structure showing flexible movements in cytosolic domains.

Title: Supplementary Movie 3

Description: Unbiased MD simulations of a symmetric model of MtCorBΔC.

Title: Supplementary Movie 4

Description: Targeted MD simulations of MtCorB TMD from inward-facing to outward-facing conformations.
